# African swine fever vaccine ASFV-G-ΔI177L induces solid protection in four-week-old piglets

**DOI:** 10.1038/s41598-025-31405-3

**Published:** 2025-12-23

**Authors:** Xuan Hanh Tran, Le Thi Thu Phuong, Nguyen Quang Huy, Do Thanh Thuy, Nguyen Van Dung, Lauro Velazquez-Salinas, Elizabeth Ramirez-Medina, Cyril G. Gay, Manuel V. Borca, Douglas P. Gladue

**Affiliations:** 1 National Veterinary Joint Stock Company (NAVETCO), Ho Chi Minh City, 70000 Vietnam; 2 U.S. Department of Agriculture, Agricultural Research Service, Beltsville, MD 20705 USA; 3 U.S. Department of Agriculture, Agricultural Research Service, Plum Island Animal Disease Center, Greenport, NY 11944 USA; 4 U.S. Department of Agriculture, National Bio-Agro Defense Facility, Agricultural Research Service, Manhattan, KS 66506 USA; 5 Present Address: Seek Labs, 350 W 800 N Suite 220, Salt Lake City, UT 84103 USA

**Keywords:** ASFV, ASFV-G-ΔI177L, Live attenuated vaccines, Vaccination in piglets, Vaccines, Virology

## Abstract

African swine fever (ASF) is an important disease of swine currently affecting pig production worldwide. Vietnam is, currently, the only country where commercial live attenuated vaccines are being freely used in the field. One of these vaccines is based in the use of the ASFV-G-ΔI177L strain, a recombinant virus developed by a partial deletion in the I177L gene from the highly virulent parental strain Georgia 2010. The commercial version of the vaccine was originally limited to use in pigs between 8 and 10 weeks of age, which significantly restricts its use. In this report, we demonstrate that pigs can be vaccinated as early as the fourth week of age, producing an efficacious immune response that fully protects the animals against the challenge with the virulent Vietnamese field strain TTKN/ASFV/DN/2019 and thus increasing the vaccine’s usage to pigs 4–10 weeks of age. Several groups of four-week-old pigs were intramuscularly (IM) vaccinated with a single dose of a commercial vaccine containing 10^2.6^ HAD_50_ of ASFV-G-ΔI177L and IM challenged 28 days later with 10^2^ HAD_50_ of TTKN/ASFV/DN/2019. All the vaccinated animals remained clinically normal after vaccination, demonstrating no presence of residual virulence of ASFV-G-ΔI177L in animals of this age. In addition, all vaccinated animals remained protected after the challenge, showing no clinical signs associated with ASF during the observational period. These results corroborate the safety and efficacy of the ASFV-G-ΔI177L vaccine strain when used in pigs as early as four week of age.

## Introduction

African swine fever virus (ASF) is a usually lethal disease affecting the production of domestic pigs worldwide^1^. ASF was geographically restricted to south Saharan Africa. However, after an initial outbreak in the Republic of Georgia in 2007, ASF quickly became pandemic, affecting Central and Eastern Europe, China, and Asia. In, 2021, ASF spread to the Caribbean region after being absent from the Western hemisphere for the last 50 years^2–4^.

The development of new ASF experimental vaccines have increased in the last years, with multiple laboratories worldwide deleting genes in ASFV genome^5^. Live attenuated vaccine candidates have been developed by deletion of ASFV genes via various methodologies of genetic manipulation^6,7^. However, despite multiple experimental vaccines, only two vaccine strains to prevent ASF are commercially available. In 2022, Vietnam authorized the commercial use of the first modified live vaccines based in the use of the ASFV-G-∆I177L^8^ and the ASFV-G-ΔMGF strains^9^. ASFV-G-∆I177L harbors a deletion in the I177L gene from the genome of the field parental strain Georgia (ASFV-G). ASFV-G-∆I177L efficiently protected pigs even when used at doses as low as 10^2^ HAD_50_ against the challenge with the virulent homologous ASFV-G as well as with Vietnamese field strains and even when using animals of different genetic backgrounds^10^. In addition, ASFV-G-ΔI177L has been shown to be genetically stable in reversion to virulence studies^11^ and to lack residual virulence even in long term studies^12^.

On initial release, the commercial version of ASFV-G-ΔI177L is recommended to be used in animals between 8 and 10 weeks of age with a regime including two sequential doses administered 21 days apart. Here we report experimental evidence to expand use of the vaccine in pigs as young as 4 weeks of age, by testing the efficacious use of this vaccine strain in four-week-old pigs and administering only one dose. Most of the animals tested with this vaccination regime developed an efficient antibody response and were protected against the challenge with a virulent Vietnamese field isolate.

## Materials and methods

### Viruses and cells

Cell cultures of peripheral blood swine macrophages (PBSM) were prepared exactly as described previously^13^ and seeded at a concentration of 1.5 × 10^6^ cells per T75 cm^2^ flask. Virus titration was performed on PBMS cell cultures in 96-well plates as previously described^14^. Presence of virus infected cells was detected by the hemadsorption (HA) of swine erythrocytes, and virus titers were calculated by the Reed and Muench method^15^. The ASFV-G-ΔI177L strain was provided by Navetco^10^. Challenge experiments were performed using the highly virulent Vietnamese field isolate TTKN/ASFV/DN/2019, obtained from the National Center for Veterinary Medicine Control (VMC) No 1 (Hanoi, Vietnam)^10^. The TTKN/ASFV/DN/2019 isolate belongs to the ASFV genotype 2 and is a derivative of the Geogia2007 strain.

### Animal experiments

All experiments were conducted using crossbreed European Yorkshire and Landrace animals sourced from Marshall BioResources. All pigs tested negative for the presence of ASF, PRRS, FMDV, CSF, PCV2,3, mycoplasma antigens and ASFV specific antibodies. Unless specifically indicated, all animal experiments were performed at the NAVETCO facility under controlled experimental conditions, following VICH guidelines for target animal safety studies: VICH GL 44 (Target Animal Safety), Biologicals, Ref. EMEA/CVMP/VICH/359665/2005 and VICH GL 41 (Target Animal Safety). Vaccine doses (always administered IM) used in these experiments are the dose for the commercial ASFV-G-ΔI177L Navetco vaccine. Challenge experiments were performed by IM inoculation of 10^2^ HAD_50_ of the virulent Vietnamese field isolate TTKN/ASFV/DN/2019 at 28 days post vaccination. Presence of ASF associated clinical signs (anorexia, depression, fever, purple skin dis-coloration, staggering gait, diarrhea, and cough) and changes in body temperature were recorded daily after vaccination and challenge. Animals presenting 3 consecutive days with rectal temperatures higher than 40 °C accompanied by at least another ASFV sign were subjected to euthanasia. Euthanasia procedures for animals requiring euthanasia are first tranquilized with Xylazine (1-2 mg/kg/IM), then anesthetized with Zoletil ^®^ 50 (1 mg/kg/IM) and later euthanized with Potassium chloride (75–150 mg/kg IV). Animal studies were in accordance with the ARRIVE guidelines.

###  Quantitative real time PCR protocol for the detection of ASFV genome

DNA extractions were performed using a Patho Gene-spin DNA/RNA Extraction Kit (Intron Biotechnology, Gyeonggi, Korea) for nasal swab samples or a QIAamp DNA Blood Mini Kit (Qiagen, Hilden, Germany) for blood samples. Detection of ASFV genome, using B646L (encoding for p72) as the target gene, was carried out using the VetMAX African SwineFever Virus Detection Kit (Thermo Fisher Scientific, Waltham, MA, USA) as previously described^16^. Samples with Ct values < 45 were considered positive.

### Detection of anti-ASFV antibodies

Presence of ASFV specific antibodies was detected by using a commercial ELISA test (Ingezim PPA, Ingenasa, Madrid, Spain) following the manufacturer’s instructions. Results were expressed as % of reactivity between 40% and 50%(positive = > 50% / negative = < 40%). Alternatively, virus specific antibodies were quantified using an in house developed ELISA as previously described^17^.

## Results and discussion

### Detection of residual virulence in four week old piglets vaccinated with ASFV-G-ΔI177L

The main objective of this work was to evaluate the efficacy of ASFV-G-ΔI177L in inducing protection against the virulent challenge in piglets vaccinated at four weeks of age. For that purpose, three similar experiments were performed independently, each consisting of two groups with five piglets in each group that were IM vaccinated with either a full (10^2.8^ HAD_50_) or half (10^2.5^ HAD_50_) dose of the ASFV-G-ΔI177L vaccine. A third group, composed of three animals, was used as mock vaccinated control group. In each experiment, all three groups were IM challenged at 28 days post vaccination with 10^2^ HAD_50_ of the Vietnamese field isolate TTKN/ASFV/DN/2019.

The presence of clinical signs associated with ASF was evaluated daily after vaccination and also after challenge until the end of the experimental periods of 26 days post inoculation (dpi) and 21 days post challenge (dpc), respectively.

**Fig. 1:**
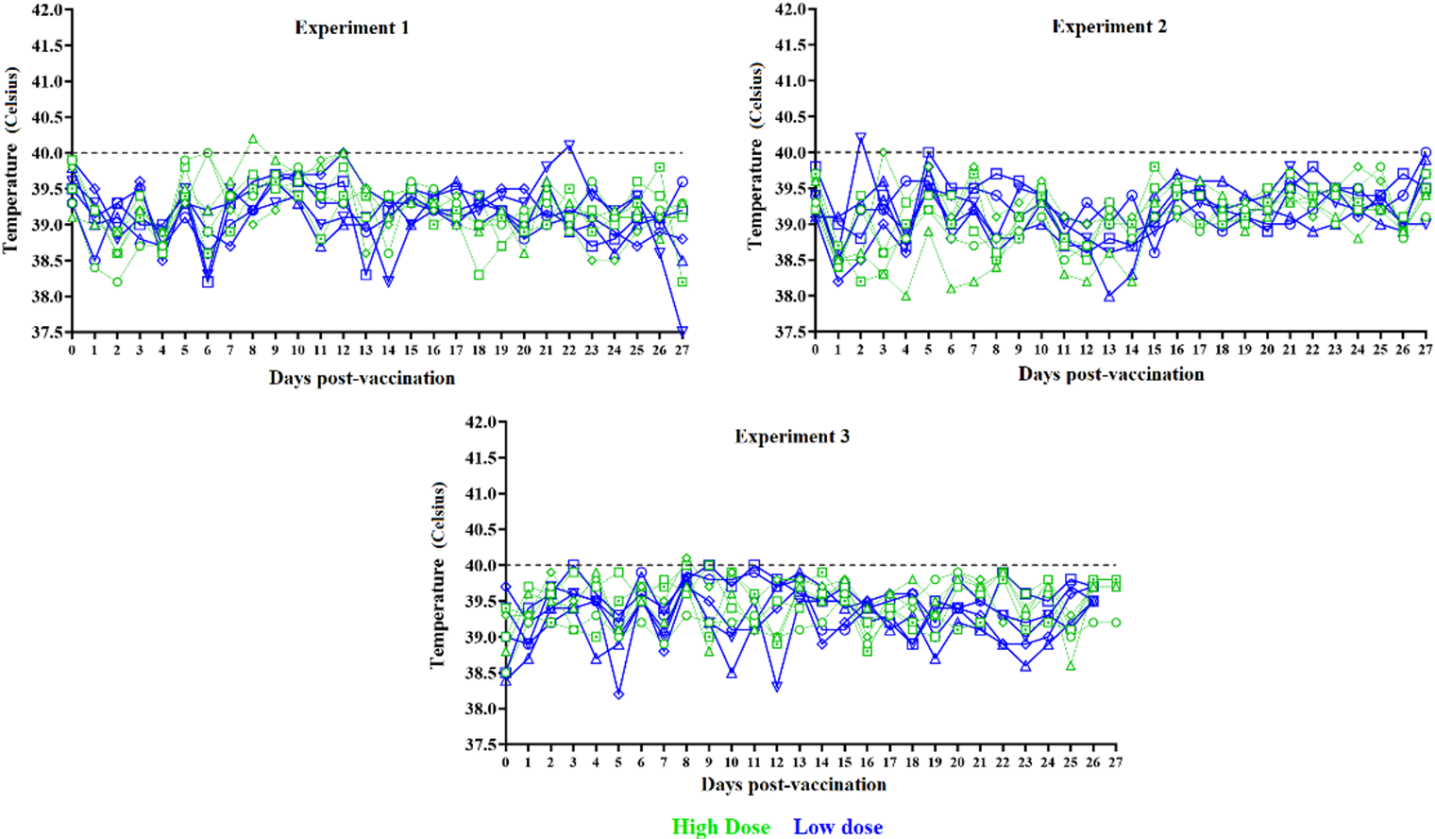
Kinetics of body temperature in animals inoculated with ASFV-G-ΔI177L. Groups (*n* = 5) of pigs were IM inoculated with either a full (10^2.8^ HAD_50_) or half (10^2.5^ HAD_50_) dose of ASFV-G-ΔI177L. Rectal temperature was recorded daily until day 26 post vaccination. The experiment was performed three times using the same experimental design. Data represent individual animals.

The clinical evaluation performed on a daily basis after vaccination demonstrated the complete absence of any significant abnormality or any clinical signs that may be associated with the disease in either of the two vaccinated groups in any of the three independent experiments (Fig. 1). The daily body temperatures in the two groups in each of the three experiments showed that with the exception of a few isolated cases, all animals presented values lower than 40 °C during the 27 days after vaccination. Therefore, administration of the commercial version of ASFV-G-ΔI177L to piglets at 4 week of age does not produce any alteration in the clinical status of the vaccinated animals.

### Presence of virus specific antibody response in four week old piglets vaccinated with ASFV-G-ΔI177L

The presence of ASFV specific antibodies was evaluated in all vaccinated animals on day 26 post vaccination by two different ELISA tests. As described in the Material and Methods, a commercial ELISA (CE in Table 1) qualifies serum samples as either positive or negative, while an in house developed ELISA^17^ (IHE in Table 1) allows the titration of antibodies in the evaluated serum samples. Taken all together, in the three experiments, 26 out 30 (87% of the animals) of the pigs vaccinated with either a full or half dose of vaccine (10^2.8^ HAD_50_) or half (10^2.5^ HAD_50_) dose of ASFV-G-ΔI177L developed ASFV specific antibodies detected by the CE test. Similarly, 24 out of 30 (80% of the animals) pigs receiving either the full or half dose of vaccine also developed virus specific antibodies, though most of them did so with low titers (between 10^− 1^ to 10^− 2^ dilution). Therefore, most of the 4 week old vaccinated pigs were able to develop ASFV specific antibodies.

**Table 1:** ASFV-specific antibodies detected by two ELISA methods in pigs after 26 days of IM receiving a full (10^2.8^ HAD_50_) or half (10^2.5^ HAD_50_) dose of ASFV-G-DI177L.

Test	Experiment 1										Experiment 2										Experiment 3									
	Full dose					Half dose					Full dose					Half dose					Full dose					Half dose				
CE	+	+	−	+	+	+	+	−	+	+	+	+	+	+	+	+	+	−	+	+	+	+	+	+	+	+	+	−	+	+
% of I	71	62	39	70	84	83	65	48	80	61	69	71	58	71	61	66	54	24	74	74	69	71	58	71	61	56	74	24	57	74
IHE	2	2	−	2	3	2	2	−	1	2	2	2	−	3	2	2	1	−	1	1	2	2	−	2	2	2	2	−	1	2

Results are presented as positive (+) or negative (-) and in percentage of inhibition (% of I) when measured with the ELISA commercial test (C) and as the log10 of the inverse of the highest serum dilution at least duplicating the OD readings of the corresponding preimmune serum when using an in house developed ELISA (IHE).

### Assessment of protection against the experimental infection in four week old piglets vaccinated with ASFV-G-ΔI177L

The evaluation of the efficacy of the ASFV-G-∆I177L inoculation in inducing protection against infection with virulent ASFV was performed using the animals previously inoculated with either a full (10^2.8^ HAD_50_) or half (10^2.5^ HAD_50_) dose of ASFV-G-ΔI177L. At day 28 post vaccination, these two groups of animals were IM inoculated with 10^2^ HAD_50_ of of the Vietnamese field isolate TTKN/ASFV/DN/2019. Along with the vaccinated animals, in each expriment, a group (*n* = 3) of naïve animlas were also challenged under the same conditions and used as controls. Animals were monitored daily for their body temperature and the appearance of clinical signs related with ASF during an observational period of 21 days (Table 2).

**Table 2:** Swine survival and fever response in pigs receiving either a full (10^2.8^ HAD_50_) or half (10^2.5^ HAD_50_) dose of ASFV-G-ΔI177L and IM challenged 28 days after vaccination with 10^2^ HAD_50_ of the Vietnamese field isolate TTKN/ASFV/DN/2019.

Experiment	Vaccine dose (HAD_50_)	Mean time to death (days ± SD)	Fever		Number of survivors/total
			Number of days to onset (days *±* SD)	Duration (days ± SD)	
1	10^2.8^	–	–	–	5/5
	10^2.5^	–	–	–	5/5
	None	8 (0.00)	4.33 (0.58)	4.67 (0.57)	0/3
2	10^2.8^	-	–	–	5/5
	10^2.5^	-	–	–	5/5
	None	7.33 (1.53)	3.66 (0.58)	4.33 (1.53)	0/3
3	10^2.8^	–	–	–	5/5
	10^2.5^	–	–	–	5/5
	None	8.3 (0.57)	4.6 (1.15)	3.6 (0.57)	0/3

(–): the event does not occur. Arithmetical average and Standard Deviation (SD) values were calculated using the Microsoft Excel program.

All vaccinated animals remained clinically normal after the challenge without showing any clinical signs associated with ASF during the observational period of 21 days post challenge. Daily rectal temperature measurements only occasionally showed values reaching 40 °C. On the other hand, all control animals developed lethal forms of ASF starting around day 3–5 post challenge, worsening quickly and being euthanized due to the severity of the clinical signs of the disease between 7 and 9 days post challenge.

Potential presence of virus in blood of vaccinated and challenged pigs was assessed at 14 and 23 dpc by PCR targeting structural protein ASFV p72. In the first experiment at 14 dpc, all 10 vaccinated animals had negative results. In the second experiment, 9 out of the 10 vaccinated pigs produced Ct values between 33.39 and 42.74, with only one of the animals receiving the low dose of vaccine being negative. In the third experiment, 9 of the 10 vaccinated pigs showed Ct values between 36.13 and 43.45, with only one of the animals receiving the lower vaccine dose having negative results, similar to what was observed in the second experiment. All vaccinated animals involved in all three experiments tested negative by PCR at 23 dpc. It should be noted that all 18 vaccinated animals testing positive at 14 dpc by p72 specific PCR were retested and all resulted negative with a in house developed PCR that specifically target the I177L^8^ indicating that virus detected at 14 dpc was only ASFV-G-ΔI177L.

## Conclusion

The results presented here indicate that the ASFV-G-∆I177L vaccine candidate can be used in inducing protection in pigs as early as four week of age. It is shown that ASFV-G-∆I177L does not produce any side effect after vaccination, showing no residual virulence in animals of this age. In addition, vaccinated pigs are solidly protected against the challenge with the virulent Vietnamese field strain even after the inoculation of only one dose of ASFV-G-∆I177L. Therefore, ASFV-G-∆I177L can be used in young animals, inducing effective protection after the administration of only one vaccine dose and as early as four weeks of age. The possibility of using the vaccine in younger animals significantly widens the period when animals can be vaccinated, reducing the period where piglets are susceptible to be become infected with ASF.

## Data Availability

Data is contained within the article.
